# Exploring Resource-Sharing Behaviors for Finding Relevant Health Resources: Analysis of an Online Ovarian Cancer Community

**DOI:** 10.2196/33110

**Published:** 2022-04-12

**Authors:** Khushboo Thaker, Yu Chi, Susan Birkhoff, Daqing He, Heidi Donovan, Leah Rosenblum, Peter Brusilovsky, Vivian Hui, Young Ji Lee

**Affiliations:** 1 School of Computing and Information University of Pittsburgh Pittsburgh, PA United States; 2 School of Information Science University of Kentucky Lexington, KY United States; 3 School of Nursing University of Pittsburgh Pittsburgh, PA United States

**Keywords:** online health community, resource sharing, link sharing, topical relevance, information seeking, ovarian cancer, user behavior modeling

## Abstract

**Background:**

Online health communities (OHCs) provide patients and survivors of ovarian cancer (OvCa) and their caregivers with help beyond traditional support channels, such as health care providers and clinicians. OvCa OHCs promote connections and exchanges of information among users with similar experiences. Users often exchange information, which leads to the sharing of resources in the form of web links. Although OHCs are important platforms for health management, concerns exist regarding the quality and relevance of shared resources. Previous studies have examined different aspects of resource-sharing behaviors, such as the purpose of sharing, the type of shared resources, and peer user reactions to shared resources in OHCs to evaluate resource exchange scenarios. However, there is a paucity of research examining whether resource-sharing behaviors can ultimately determine the relevance of shared resources.

**Objective:**

This study aimed to examine the association between OHC resource-sharing behaviors and the relevance of shared resources. We analyzed three aspects of resource-sharing behaviors: types of shared resources, purposes of sharing resources, and OHC users’ reactions to shared resources.

**Methods:**

Using a retrospective design, data were extracted from the National Ovarian Cancer Coalition discussion forum. The relevance of a resource was classified into three levels: relevant, partially relevant, and not relevant. Resource-sharing behaviors were identified through manual content analysis. A significance test was performed to determine the association between resource relevance and resource-sharing behaviors.

**Results:**

Approximately 48.3% (85/176) of the shared resources were identified as relevant, 29.5% (52/176) as partially relevant, and 22.2% (39/176) as irrelevant. The study established a significant association between the types of shared resources (*χ*^2^_18_=33.2; *P*<.001) and resource relevance (through chi-square tests of independence). Among the types of shared resources, health consumer materials such as health news (*P*<.001) and health organizations (*P*=.02) exhibited significantly more relevant resources. Patient educational materials (*P*<.001) and patient-generated resources (*P*=.01) were more significantly associated with partially relevant and irrelevant resources, respectively. Expert health materials, including academic literature, were only shared a few times but had significantly (*P*<.001) more relevant resources. A significant association (*χ*^2^_10_=22.9; *P*<.001) was also established between the purpose of resource sharing and overall resource relevance. Resources shared with the purpose of providing additional readings (*P*=.01) and pointing to resources (*P*=.03) had significantly more relevant resources, whereas subjects for discussion and staying connected did not include any relevant shared resources.

**Conclusions:**

The associations found between resource-sharing behaviors and the relevance of these resources can help in collecting relevant resources, along with the corresponding information needs from OvCa OHCs, on a large scale through automation. The results from this study can be leveraged to prioritize the resources required by survivors of OvCa and their caregivers, as well as to automate the search for relevant shared resources in OvCa OHCs.

## Introduction

### Background and Motivation

Ovarian cancer (OvCa) affects approximately 22,000 women per year in the United States [1-3] with a 70% recurrence rate [4]. Survivors of OvCa are individuals diagnosed with cancer irrespective of their state of disease [5]. They typically receive intensive oncological treatment, which has adverse effects on their quality of life [6-10]. Both survivors of OvCa and their caregivers require support and have various information needs throughout the course of OvCa [11,12]. Health care providers try to address their common information needs through standardized patient and caregiver educational materials; however, these materials may lack information to address both survivors’ and their caregivers’ unique and dynamic information needs [13,14].

To meet their unique information needs, a growing number of survivors of OvCa and their caregivers generally seek support from online health communities (OHCs) on a regular basis. OHCs enable these individuals to connect and exchange information with other individuals with similar experiences [15-18]. OHCs specific to gynecological cancer also provide a platform where women with OvCa can freely share their experiences and feel a strong sense of belonging [19]. Owing to their powerful communal nature, OHCs could offer survivors of OvCa and their caregivers an opportunity to exchange information individualized to their needs. This exchange of information often leads to resource sharing among users in the form of web links [17,18]. The resources shared among OHC users can serve as educational materials that address their unique information needs. These shared resources can potentially benefit survivors and caregivers by helping them acquire knowledge about different aspects of the disease, including but not limited to treatment, diagnosis, and disease management.

Despite the benefits of shared resources, some important questions arise, given that OHC users are health consumers and might not be health experts: which resources shared by the OHC peers are relevant to the information needs of survivors of OvCa and their caregivers, and what aspects of resource sharing can help us determine resource relevance? Previous research examined health literacy in OHCs and revealed that most of the content is generated by users with underdeveloped skills in validating information sources and navigating the internet [20]. Therefore, users need help in finding the relevant resources generated or shared in OHCs [21]. Motivated by this, the objective of this study is to examine the connections between users’ resource-sharing behaviors and the relevance of shared resources. The outcomes can help future research locate relevant resources that are helpful in educating survivors and caregivers on OvCa OHCs. This study is part of an ongoing project, Health e-Librarian with Personalized Recommender (HELPeR), which aims to recommend personalized, relevant information resources to survivors of OvCa and their caregivers (HELPeR study 1R01LM013038-01A1). The ultimate goal of HELPeR is to improve the quality of user-focused recommendations in all aspects of OvCa care.

Most previous studies examined resource sharing in OHCs [22,23], although little attention has been paid to understanding if these resources are relevant to user information needs. Few studies have examined the quality and relevance of user-generated data on OHCs [24-27]; however, these studies are based on the content of the user post and do not address the quality of shared resources. This study fills this gap by exploring the relevance of the shared resources. This study extends previous studies by determining the relevance of shared resources and post content. Examining the relevance of resources will reveal what resources can help fulfill the information needs of survivors of OvCa and caregivers. Resource relevance has multiple dimensions, including topical relevance, readability, trustworthiness, timeliness, and clinical validity [28,29]. This paper considers topical relevance, which defines whether the content addresses the information needed [28]. A resource is relevant if its content addresses the information needed by the user; otherwise, the resource is irrelevant. In the rest of the paper, the words *relevance* and *topical relevance* are used interchangeably.

User behavior has been substantially explored in the context of search engines and recommender systems [30-33]. For example, users’ seeking behaviors are examined to improve search quality by determining the relevance of a search result against users’ information needs [30,31,34]. User behavior can help provide 2 types of user feedback. *Explicit* feedback is where users themselves provide feedback about the relevance of an item (eg, liking a search result). On the other hand, implicit feedback is obtained without user intervention (eg, by tracking the dwell time on a search result page). Recently, user behavior has also been used in web-based community research [24,35]. Wanas et al [24] used web-based community–specific user behaviors, including the presence of quotations in a post (implicit) and the number of replies to a post (implicit), along with other features to train a post quality scoring algorithm. Explicit feedback, including post likes [35], and implicit feedback, including participant reputation [36], were also used to determine the relevant posts in a thread in a social media forum. Differing from previous studies, this study explores resource-sharing behaviors pertaining to OHC users to determine shared resource relevance. In OHCs, resource-sharing behaviors are examined to determine how OHC members engage with shared resources [22,23]. Zhang and Sun [22] examined the purpose of resource sharing in a web-based diabetes forum to reveal the support that shared resources provide. Nathan et al [23] studied the types of resources shared in an OHC and OHC users’ *like* reaction on WebMD threads [37] to reveal the types of resources trusted by OHC users. Although resource-sharing behaviors have been studied in OHCs, there is no study on whether these resource-sharing behaviors can determine the relevance of shared resources. Given the dearth of research in this area, the purpose of this study is to examine (1) the relevance of resource sharing on an OvCa OHC and (2) users’ resource-sharing behaviors associated with shared resource relevance in an OvCa OHC. Examining both resource relevance and resource-sharing behaviors provides insights into which user behaviors are associated with relevant and irrelevant resources.

### Objectives

**Figure 1:**
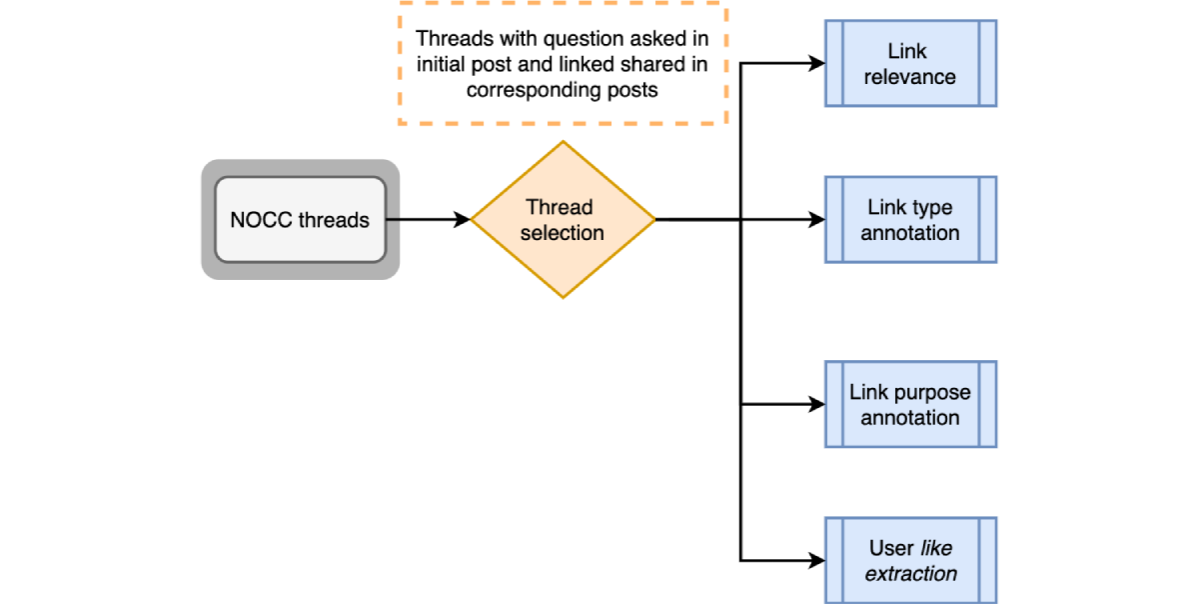
Workflow of the study. NOCC: National Ovarian Cancer Coalition.

Figure 1 provides the overall description of our study design. This study was a descriptive analysis of the OvCa OHC threads. Three aspects of resource-sharing behaviors were considered: type of resource shared, purposes of sharing a resource, and OHC users’ *like* reactions to the resource shared. Types of shared resources and the purpose of sharing resources provide implicit user feedback, as they do not explicitly reveal users’ interests or likes on a resource. An OHC user’s *like* reaction on the shared resource provides explicit user feedback, where the user explicitly reveals their interest in the shared resource. This study investigates the following three research questions (RQs) to explore resource relevance along with resource-sharing behaviors:

- RQ1: what is the relationship between the type of resources shared and the relevance of these resources in an OvCa OHC?
- RQ2: what is the relationship between the purpose of sharing resources and the relevance of these resources in an OvCa OHC?
- RQ3: what is the relationship between OHC users’ reactions to comments on the shared link and the relevance of these resources in an OvCa OHC?

## Methods

The study was performed on the National Ovarian Cancer Coalition (NOCC) forum. To address the RQs, we first determined the relevance of the shared resources and later used different resource-sharing behaviors to calculate their association with relevance using a chi-square test.

### Data Source and Collection

For OvCa OHC data, we relied on NOCC [38]. NOCC is a subcommunity of the Cancer Connect Community [39], which brings together survivors of OvCa and caregivers. NOCC users start threads in seeking information, receiving a second opinion, sharing experience, and receiving emotional support, whereas other participants provide support by replying to these threads in the form of comments. Forum users also express gratitude toward posts and comments using the like button. The NOCC is a patient-oriented community in which moderators are also survivors of OvCa or caregivers. We selected the NOCC because of its two unique properties:

1. It is an OvCa-specific community, which is a rare cancer with less exposure or awareness among general survivors of cancer and caregivers.
2. OvCa is a women-only cancer; therefore, the platform allows for the free exchange of information and resources with other individuals with similar experiences, where OHC users have developed a sense of community and connection [19].

**Figure 2:**
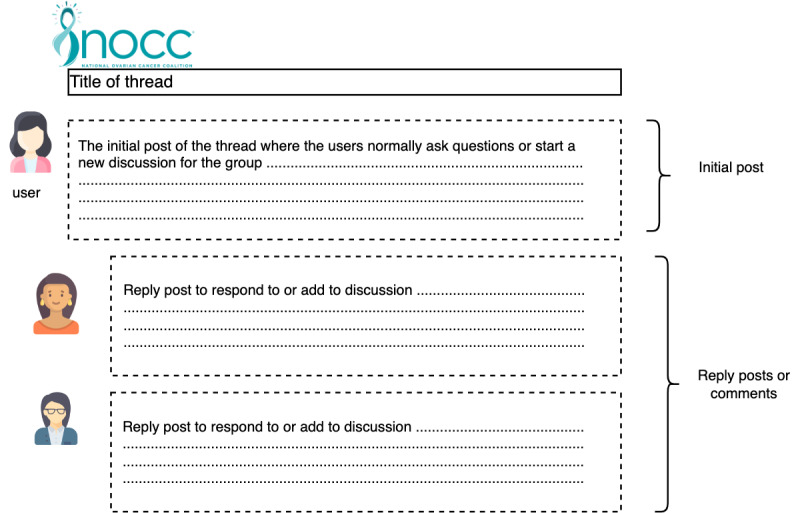
A typical National Ovarian Cancer Coalition thread component, which includes the thread poster, title of thread, initial post, reply posts, and *like* button. The actual content of the thread was removed for privacy of National Ovarian Cancer Coalition users. The purpose of this figure is to provide readers with a basic understanding of communication patterns on this forum.

NOCC is not a public community; therefore, we obtained permission from the institutional review board to collect and analyze the forum content. We collected data available from June 2010 to December 2020. Each thread comprises an initial post and replies to comments. For each thread, the following information was recorded: the title of the thread, initial post content, poster’s name, all comments on the post, comment users’ names, number of likes on comments, number of likes on posts, users who liked, time of posts, and time of comments. Figure 2 shows an example of a NOCC thread and its different components. The actual content of the post was removed to better show the basic structure of the thread and ensure patient privacy. Each thread is initiated by a NOCC user, which includes the title of the thread and an initial post. The initial post is followed by comments and replies from the forum users. Comments or reply posts are where the resources are shared in response to the information needed in the initial post.

The data for analysis were deidentified to remove participant information from the initial posts and all comments. From the 909 threads, we selected 105 (11.6%) threads for this study, as explained below:

1. First, we filtered posts containing advertisements from health organizations. These threads included advertisements such as survey enrollment, product advertisements, and monthly updates from the NOCC moderator.
2. Then, of the 909 threads, 495 (54.5%) threads were selected in which the initial post contained a question. For simplicity, in the following sections, we would refer to this data set of 105 threads as NOCC question threads.
3. From the 495 selected threads, we further examined 105 (21.2%) threads where users shared resources (URLs) in their reply comments.
4. Links were extracted from 105 threads using regular expressions [23]. We found 176 links shared among these 105 threads.
5. For our final data set, we assembled 176 post–comment pairs, where each post had a question, and each comment contained a shared link. Thereafter, we will call this data set with 176 post–comment pairs the NOCC shared resource (NOCC-RS) data set.

Manual content analysis was performed on NOCC-RS to annotate relevance, types of resources, and purpose of sharing resources (Figure 1). Each annotation procedure was performed separately to ensure that one annotation did not influence the other. To report the quality of each annotation, we calculated the interrater reliability score using Cohen κ [40]. Cohen κ (equation 1) is a widely accepted measure for ensuring the quality of annotator agreement and is more robust than calculating percentage agreement [40]. A percentage agreement of ≥0.85 [41] and a Cohen κ coefficient of ≥0.5 [42] are acceptable quality for annotations. As a result, an acceptable κ measure was obtained:



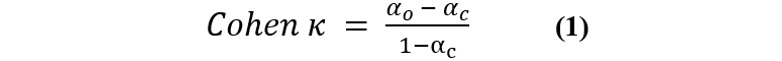





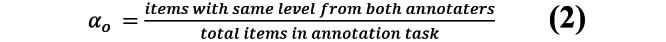





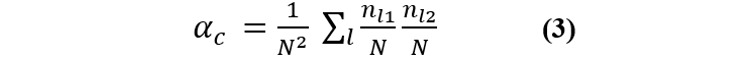



Here, *α_o_* is the probability of an item receiving the same code from both annotators, and *α_c_* is the probability of agreement occurring by chance. N is the total number of items for annotation, and *n_li_* is the number of times an annotator *i* predicted label *l*.

All annotators met every week to decide the coding schema for each annotation, discuss disagreements on overlapping samples, and calculate the κ score. In the following sections, each annotation process is discussed in detail, along with the coding schema.

### Resource Relevance Annotation

To assess the relevance of each resource shared for the corresponding information needed (ie, the question in the initial post), we developed a coding scheme that classified the resources into three categories: relevant, partially relevant, and irrelevant. For each resource, annotators, VH and YC, first checked the initial post that contained the question and then read the comment post that contained the link. Relevance was judged based on the topical relevance between the link and the question asked in the corresponding thread. The study engaged two domain experts to accomplish this task: VH was a nurse, and YC was a researcher focused on the needs of survivors of OvCa and caregivers. Initially, the annotators started with a binary coding scheme: relevant and irrelevant. Later, after discussion among annotators, they found that there were many resources that did not provide the original information needed by the user but were still helpful to the user. Thus, although partially relevant resources did not answer the question, they were either usable for users, given their information needs, or helpful to the user to reach the relevant resource. This resulted in the 3 categories described in Textbox 1. Textbox 2 provides examples of all 3 categories from the NOCC forum post. The interrater agreement between the 2 annotators is Cohen κ=0.65, calculated on 39.8% (70/176) data overlap, with a substantial agreement of 81%.

**The classification scheme for resource relevancy with description and corresponding example.**

**Code and description (all relevance annotations were based on topical relevance)**

- Irrelevant
  The information provided through the resource does not address the corresponding question asked.
- Partially relevant
  The information provided through the resource does not provide a direct answer to the corresponding question but can either provide some related information to find relevant information or is useful to the user.
- Relevant
  The information provided through the resource directly addresses the corresponding question and provides an answer to the corresponding question.

**Example posts (some information is removed for anonymization).**

**Initial post with a question**

- “I was diagnosed with ovarian stage 3c—background information—My doctor wants to add Avastin to my next 3 rounds of chemo. I am worried about adding it because of all the side effects I already had a reaction to the carbo once and that was very bad. Do you know anything about the side effects of Avastin?”

**Relevance and comments with a shared resource**

- Relevant resource: “Avastin definitely plays a major role in both treatment and maintenance therapy for a number of cancers. About Avastin; news.cancerconnect.com/treatment-care/answers-to-faq-s-about-avastin/”
- Partially relevant resource: “Hi XXX, treatment decision-making can be so difficult. Good for you for looking at all your options. Have you had a second opinion at another large cancer center? Asking your doctors about the risks and benefits of each treatment option is important. The NCCN patient guidelines for ovarian cancer might also be a helpful resource for you (www.nccn.org/patients/guidelines/ovarian/index.html). Hope this helps and keep us posted!”
- Irrelevant: “If you want to discuss this more and want to connect, please connect to my blog: http://xxx.blogspot.com”

### Resource Type Annotation

To answer RQ1, the shared resources were categorized. Each resource was categorized based on the *domain* and *content* of the links. For domain name–based categorization, we relied on the top-level domain (TLD) of the URL, as in the study by Nathan et al [23]. Domain names are designed to represent websites distributed among various hosts and network systems, with a string of characters usually separated by dots as their structure. The TLD is the last part of the domain name of US websites. If a domain name is outside the United States, its TLD is the second to last part of the URL. From the TLD, one can determine the entity, administrator, and intended use of a website [43]. For example, the TLD of *ncbi.nlm.nih.gov* is *.gov*, indicating that the website belongs to a governmental entity, and that of *ovarian.org* is *.org*, indicating that it is an organization website. This study adopted 6 TLDs, including *.com*, *.edu*, *.org*, *.net*, *.io*, and *.gov*.

To move beyond simple domain name–based analysis, we manually examined each link and classified the shared resources into content-focused categories. Initially, two coders (KT and YC) separately coded the links using the coding scheme mentioned in [44], which is specifically used for the classification of health domain webpages during the consumer search process. During the subsequent debriefing, the discussion among coders about disagreements led to the refinement of the original categories. Two new categories were introduced—nonhealth articles and patient educational resources—which were missing from the previous study. Table 1 provides the final 10 types used to classify resources. It is assumed that the links belonging to each category have similar types of content and are for similar consumers. The interannotator agreement between 2 annotators was Cohen κ=0.8, calculated on 19.3% (34/176) data overlap.

**Table 1:** Coding scheme for resource types with description and corresponding example.

Code	Description	Example domains
Health articles	A link containing focused information about one specific health topic with content written for health consumers in mind; this could include health articles, health expert blogs, and health topic information websites	Cancer.net [45] Med-Health.com [46]
Health news	A webpage presenting health news; this could include news about findings in research, treatment results, and updates on medications and clinical trials	CancerConnectNews [39] Medicalexpress [47]
Patient educational resource	Resources provided by government and cancer organizations, including patient guidelines, factsheets, and patient booklets	Cancer.gov [48] NCCN.org [49]
Academic literature	Research articles and clinical trial articles	NCBI.gov [50] Eurekalert.org [51]
Web-based social groups	A link containing user discussions and posts on web-based communities, question answering forums, and social networking sites	NOCC.ovarian.org [52] CSN.Cancer.org [53] Facebook [54]
Health organizations	A link referring to the home page of a health organization, medical school, nonprofit institute, or government website	Ovarian.org [38] Dana-Farber.org [55]
Patient blogs	Patient- or caregiver-generated personal websites and blogs	xxx.blogspot.com
e-Commerce	Online shopping sites and product promotion/advertisement web pages	Omiana [56] 100percentpure [57]
Videos	Links to video content	YouTube [58]
Nonhealth articles	Shared content outside of the health domain	Lawfirm [59] Wikipedia [60]

### Resource Purpose Annotation

The purpose of a link refers to the role the link serves in a post [22]. Zhang et al [22] unveiled the relationship between the type of forum user (frequent vs occasional contributors) and the purpose of their link-sharing behavior. The coders started with the coding schema of Zhang et al [22], which defined six roles of links shared in the initial posts: *providing additional reading*, *supporting arguments*, *subjects for discussion*, *recommendations for peers*, *the source of a post*, and *asking for help*. As coding proceeded, we removed two categories that we considered inapplicable to the link-sharing purpose in the comments (*recommendation for peers* and *asking for help*), and we added two new categories: *pointing to resources* and *staying connected*. The coding scheme includes *providing additional readings*, *supporting arguments, subjects for discussion*, *pointing to resources*, and *staying connected*. Table 2 presents the final definition of each purpose and an example comment with a URL link.

Resource purpose annotation was performed independently of resource relevance annotation and only by reading the comment and ignoring the initial post. Two coders independently annotated the role of the shared link with a 34.1% (60/176) overlap of comments between them. The final agreement after the second round of annotation was 93%, with Cohen κ=0.88, which indicates a substantial agreement. After addressing all the disagreements between the 2 coders, KT proceeded to code all the remaining comments.

**Table 2:** Coding scheme for link-sharing purposes with description and corresponding example.

Code	Description	Example (anonymized or rephrased)
Providing additional readings	The information provided through the link provides reading materials to answer the corresponding questions.	“Olaparib is a PARP inhibitor. Are you platinum-sensitive and do you have a BRCA mutation? If you do olaparib works well. Here is a great article www.targetedonc.com/publications/targeted-therapies-cancer/2017/2017-august/the-current-status-of-parp-inhibitors-in-ovarian-cancer”
Pointing to resources	The information provided through the link does not provide a direct answer to the corresponding question but can provide some related information to search for relevant information or is useful to the user; for example, link to generic OvCa information, OvCa resource listing, and clinical trial search engine.	“SOOOOOOOOOOOOOO much interesting data in here—stuff we will benefit from! Yeehaw! news.mit.edu/search?keyword=Koch+Cancer+Center”
Supporting argument	The information provided in the comment directly addresses the users’ information needs, whereas the link acts as evidence to support the facts mentioned in the comment.	“Yes, the PARP drugs seem to show promise with platinum resistance as well. news.cancerconnect.com/zejula-in-combination-with-keytruda-appears-promising-in-patients-with-platinum-resistant-refractory-ovarian-cancer/ Best XXX”
Staying connected	The link is provided for the advertisement of a personal blog, providing an email address, or connecting to an existing ovarian group.	“It is good to hear about another MMMT survivor. There seem to be so few of us because it is such an aggressive cancer cell. If you would like to connect with me more, I am at XXX@gmail.com (personal email), or XXXblogspot.com (personal blog). XXX, thank you for your kind wishes.”
Subject for discussion	“The link content is the topic that the replier wants to discuss.”	“What do you know about CART- T Immunotherapy? cancerresearch.org/immunotherapy/cancer-types/ovarian-cancer”

### User Reaction to Shared Resources

OHC websites usually provide ways for users to provide feedback (liking, disliking, and helpfulness) on posts and comments. NOCC offers its users a *like* button that can be used to display gratitude and other positive feelings about a post or comment. In modern recommender systems, signs of user appreciation such as thumbs-up and likes are signs of item relevance for the user and form the main source of knowledge for recommendations [32]. The motivation for RQ3 was to reassess this assumption in the context of an OHC and determine whether *like* reactions of OHC users on comments that contained shared resources could be used as a sign of relevance to cross-recommend *liked* resources and to serve as a *gold standard* for resource relevance studies. The *like* reactions were explored in two ways: first, *like* reaction from the user who asked the question in the initial post and second, *like* reactions from all peers on NOCC. Our hypothesis is that as the resource is shared for the information needed from the thread initiator, the *like* from this user might be a good indicator of the relevance of a resource.

### Ethical Approval

NOCC is not a public community; therefore, we obtained permission from the institutional review board to collect and analyze the forum content. Ethical approval for the study was granted in June 2021 by the Institutional Review Board of University of Pittsburgh (STUDY21050190). The institutional review board determined that the proposed activity is not research involving human subjects as defined by Department of Health and Human Services and Food and Drug Administration regulations.

## Results

### Overview

We obtained all threads from a period of 10 years from the NOCC, which is a well-known site for patients with OvCa. OvCa is a rare cancer; therefore, the NOCC had 909 threads from a period of 10 years of data collection. Furthermore, from the 909 threads, we obtained 105 (11.6%) threads with an information need (NOCC question threads), where 176 links were shared in the comments. These 176 shared links, along with the initial posts and comments with links, formed our NOCC-RS data set.

In the following sections, first, the statistics on resource relevance are presented, followed by a discussion of the association between resource relevance and resource-sharing behaviors.

### Resource Relevance

There were 85 relevant, 52 partially relevant, and 39 irrelevant resources. The relevance distribution indicates that 48.3% (85/176) of all shared links lead to resources that are relevant to the needs expressed in the original post. Furthermore, we observed that out of 105 threads, only 53 (50.5%) were answered by sharing at least one relevant resource. Of the remaining 52 posts, 48 (92%) obtained no relevant resources but ≥1 partially relevant resource. Finally, 3.8% (4/105) of posts did not receive any relevant or partially relevant resources in response.

### Resource Type

#### Resource Type Based on TLD

The most frequent TLD was *.com*, which covers 56.3% (51/176) of all shared resources (eg, *cancerconnect.com*, *youtube.com*, and *xxx.blogspot.com*), followed by *.org* (eg, *nccn.org*, *ovarian.org,* and *dana-farber.org*), *.gov* (eg, *cancer.gov*, *ncbi.nlm.nih.gov*, and *nccih.nih.gov*), *.edu* (eg, *harvard.edu*, *mit.edu*, and *vcu.edu*), *.io* (eg, *mavendoctors.io*), and *.net* (eg, *med-health.net* and *cancer.net*). We merged the remaining 2 TLDs together, which were *.me* and *.nz*, and were shared only once. Table 3 provides details on the number of links shared in each TLD and percentage of relevant resources.

To answer RQ1, we examined the association between TLDs and the relevance of a resource. The chi-square test of independence was performed on two categorical variables: TLDs (*.com*, *.gov*, *.org*, *.edu*, *.io*, and *.net*) and relevance (relevant, partially relevant, and irrelevant). The results indicated no association between TLD and relevance (*χ*^2^_12_=19.2; *P*=.10).

**Table 3:** Top-level domain (TLD)-based distribution of shared resources and percentage of relevant resources (N=176 links).

TLD	Links, n (%)	Relevant resources (n=85), n (%)	Partially relevant resources (n=52), n (%)	Irrelevant resources (n=39), n (%)
*.com*	99 (56.3)	50 (58.8)	27 (51.9)	22 (56.4)
*.org*	45 (25.6)	20 (23.5)	16 (30.8)	9 (23.1)
*.gov*	16 (9.1)	9 (10.6)	5 (9.6)	2 (5.1)
*.edu*	8 (4.5)	2 (2.4)	2 (3.8)	4 (10.3)
*.io*	3 (1.7)	2 (2.4)	1 (1.9)	0 (0)
*.net*	3 (1.7)	2 (2.4)	0 (0)	1 (2.6)
Other	2 (1.1)	0 (0)	1 (1.9)	1 (2.6)

#### Resource Type Based on Content

Table 4 provides the distribution of resources based on content type, whereas Table 1 shows an example of each resource type. Health news and health articles were the topmost shared types of resources and together accounted for 42% (74/176) of the links shared. These types were closely followed by health organizations and patient educational resources. The videos were shared in approximately 4.5% (8/176) of cases and included discussions by health experts (OncLive TV [61]), patient experiences, and other emotional support videos (relaxing music). NOCC peers also shared health organizations’ websites to fulfill information needs related to physician listings, funding institutes, and nearby nonprofit organizations. Web-based social groups were shared most of the time to point to similar previous discussions in the same OHC or another OHC. NOCC users shared their own blogs and their life journeys with their peers. Patient blogs were shared so that other OHC users could contact them, whereas commerce websites were used to share organic cosmetic products or clothing for patients with cancer.

**Table 4:** Resource type-based distribution of shared resources and percentage of relevant resources (N=176 links).

Resource type	Links, n (%)	Relevant resources (n=85), n (%)	Partially relevant resources (n=52), n (%)	Irrelevant resources (n=39), n (%)
Health news	38 (21.6)	23 (27.1)	12 (23.1)	3 (7.7)
Health articles	36 (20.5)	20 (23.5)	11 (21.2)	5 (12.8)
Health organizations	21 (11.9)	12 (14.1)	6 (11.5)	3 (7.7)
Web-based social groups	20 (11.4)	8 (9.4)	6 (11.5)	6 (15.4)
Patient resources	18 (10.2)	5 (5.9)	11 (21.2)	2 (5.1)
E-commerce	12 (6.8)	6 (7.1)	1 (1.9)	5 (12.8)
Academic literature	11 (6.3)	8 (9.4)	2 (3.8)	1 (2.6)
Patient blogs	9 (5.1)	2 (2.4)	3 (5.8)	4 (10.3)
Video	8 (4.5)	0 (0)	0 (0)	8 (20.5)
Nonhealth articles	3 (1.7)	1 (1.2)	0 (0)	2 (5.1)

To answer RQ1, the distribution of resource relevance was checked for each resource type. Table 4 provides details of the distribution of these resources. Table 4 shows that most of the relevant resources came from health news and articles, followed by health organizations. It was also interesting that the fraction of relevant resources within the category was the highest for shared academic articles. To answer RQ1, we performed a chi-square test of independence between resource relevance and resource types. We found a significant association between resource relevance and resource type (*χ*^2^_18_=33.2; *P*<.001). Furthermore, we applied the chi-square test of goodness of fit for each resource type. The results indicated that health news (*χ*^2^_2_=22.4; *P*<.001), health organizations (*χ*^2^_2_=6.0; *P*=.02), patient educational materials (*χ*^2^_2_=7.0; *P*<.001), and academic articles (*χ*^2^_2_=7.8; *P*=.01) were not equally distributed among relevant, nonrelevant, and partially relevant resources.

### Resource Purpose

**Figure 3:**
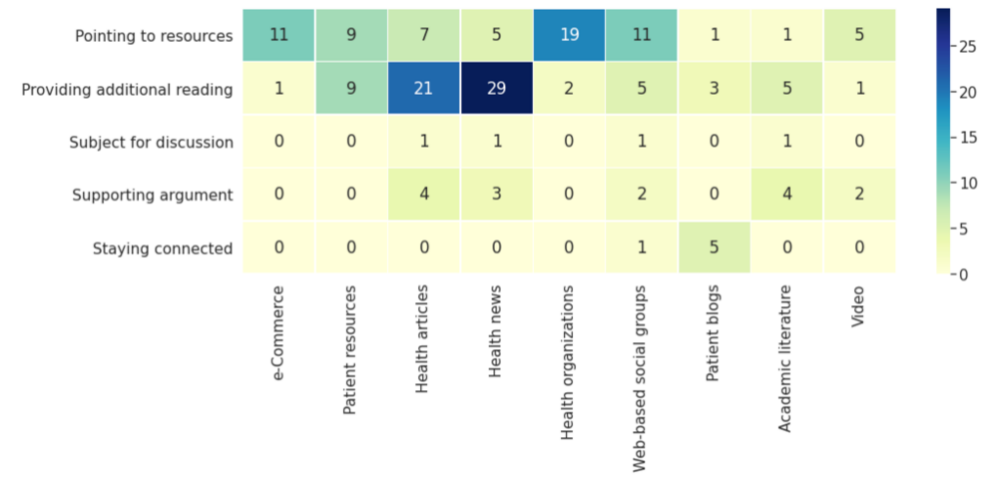
Number of different types of shared resources within each purpose.

Table 5 shows the distribution of the purposes of resource sharing. Most of the resources were shared to provide additional readings and point to resources. A much smaller proportion of the resources was shared to provide supporting arguments and subjects for discussion and to stay connected. Figure 3 shows the distribution of resource types in each of the purposes of sharing resources. It can be observed that providing additional readings can be achieved by sharing every resource type except videos. NOCC users found most of the additional readings from health articles and health news. Pointing to resources came mostly from health organizations. Academic literature was mostly shared to provide additional reading and supporting arguments.

Table 5 shows the percentage distribution of relevance for each sharing purpose. It was observed that supporting arguments resulted in the highest percentage of relevant documents, followed by providing additional readings and pointing to resources. No relevant documents were found in the roles of staying connected and subjects for discussion. The category of staying connected had some partially relevant documents; these were the cases when the initial post users’ information needs indicated an interest in connecting with patients and caregivers with similar experiences. The chi-square test of independence between resource relevance and resource-sharing purposes showed a significant association between both variables (*χ*^2^_8_=21.1; *P*<.001). Furthermore, we applied the chi-square test of goodness of fit for each resource type. Providing additional readings (*χ*^2^_2_=22.9; *P*=.01) and pointing to resources (*χ*^2^_2_=7.7; *P*=.03) were not equally distributed among relevant, nonrelevant, and partially relevant resources. This indicates that these behaviors can be used to differentiate relevant and irrelevant documents.

**Table 5:** Purpose-based distribution of shared resources and percentage of relevant resources (N=176 links).

Purpose of shared resource	Links, n (%)	Relevant resources (n=85), n (%)	Partially relevant resources (n=52), n (%)	Irrelevant resources (n=39), n (%)
Providing additional readings	84 (47.7)	43 (50.6)	26 (50)	15 (38.5)
Pointing to resources	67 (37.6)	32 (36.8)	21 (40.4)	14 (35.9)
Supporting argument	13 (7.3)	10 (11.5)	2 (3.8)	1 (2.6)
Subject for discussion	6 (3.4)	0 (0)	0 (0)	6 (15.4)
Staying connected	6 (3.4)	0 (0)	3 (5.8)	3 (7.7)

### User Reaction to Shared Resources

#### Overview

In this section, we examine the *like* reactions of the forum users to a post in the thread and its connection to the information *value* of the post. This analysis is important to assess whether the number of *likes* from the community could be considered as a sign of a post’s *value* so that posts with many *likes* could be promoted and recommended as valuable. Table 6 arranges the *like* statistics for different groups of posts in order of general increase of their value. We considered comments with links and comments to a post started by a question as potentially more valuable than average comments, as a link could provide valuable information, and a comment to a question is likely to contain a valuable answer. Comments with both properties (posted in response to a question and has a link) should be more valuable than comments with only one of these properties. To examine these *most valuable* comments in more detail, we selected 105 threads where a question was asked in initial posts and links were shared in comment posts. In Table 6, these threads are referred to as *filtered* threads. Arguably, the peak value is reached in comments within the threads that have links that are judged to be relevant to the question by the annotators. It should be noted that just the fact that a post has a link and is posted in response to a question does not assure that the link is relevant: only approximately 48.3% (85/176) of these links are relevant.

**Table 6:** Details of OHC^a^ user reactions on comments with shared resources^b^.

Comments	Relevance	Number of threads	Number of comments	Likes on comments		
				All NOCC^c^ users		Users who started the thread, n/N (%)
				Values, n (%)	Likes, mean (SD)	
All comments (909 threads)	—^d^	909	14,814	11,853 (80.01)	2.95 (2.41)	283/14,814 (1.9)
Comments with links	—	187	487	374 (76.83)	2.34 (2.01)	8/487 (1.6)
Comments in NOCC-QT^e^	—	435	6063	4382 (72.27)	2.55 (2.47)	119/6063 (2)
Comments in NOCC-RS^f^	—	105	176	110 (63.21)	1.47 (1.69)	7/176 (2.8)
Comments in NOCC-RS	Irrelevant	21	39	23 (58.82)	0.98 (1.31)	2/39 (2.9)
Comments in NOCC-RS	Partially relevant	37	52	27 (52.83)	1.41 (1.86)	1/52 (1.8)
Comments in NOCC-RS	Relevant	57	85	60 (70.93)	1.74 (1.71)	3/85 (3.5)

^a^OHC: online health community.

^b^Filtered threads are 105 threads considered in this study where a question is asked in initial posts and links are shared in comments posts.

^c^NOCC: National Ovarian Cancer Coalition.

^d^Not available.

^e^NOCC-QT: National Ovarian Cancer Coalition question threads.

^f^NOCC-RS: National Ovarian Cancer Coalition shared resource.

#### Reaction From Thread Initiator

We started by examining the *like* reactions from the user who started the thread with a question, as shown in the last column of Table 6. As the data show, the assumption that the *likes* of the target user (who wants an answer) reflect the value of the post is correct: the fraction of *liked* posts increases as we go down the table. The assumption that the target user will have a stronger *like* reaction to relevant documents shared in response to the original post is generally correct. The proportion of target user likes for *any* response to a question (119/6063, 2.04%) is higher than the number of their *likes* of an arbitrary post (283/14,814, 1.91%). The proportion of likes for a response to a question with links is even higher (7/176, 2.8%), and the proportion of likes on responses with *relevant* links (3/85, 4%) is the highest overall, approximately twice as high as an average post with a resource link (8/487, 1.6%). Unfortunately, even for relevant links, the proportion of cases in which the post initiator likes a comment to their post is very low. Although these likes follow the expected trend, their low proportion makes it impractical to use the *like* behavior of the target user as a source of data to distinguish and recommend relevant documents. To examine whether the *like* behavior is associated with the relevance of a shared resource, we performed a nonparametric Kruskal-Wallis test (*H* test). The *H* test was selected as the data were not normally distributed, and the *H* test was performed to compare likes by thread initiators on filtered threads. Although the percentage of likes was higher for comments with relevant resources, there was no significant difference (*H*=0.073; *P*=.70) between likes on comments with shared links and comments with shared relevant links.

#### Reaction From the Community

If we consider the whole community (ie, the *like* reaction of all forum users), the *coverage* of comments with likes remarkably increases. Although only 1.91% (283/14,814) of all comments were *liked* by the originating user, 80.01% (909/14,814) of comments received at least one like from the whole community, with 2.95 likes per comment on average. However, the connection between the *likes* and the information *value* of the post surprisingly goes in the opposite direction. Although the proportion of likes from the target user *increases* as we go down the table to more *valuable* posts, the proportion of community likes *decreases*. Instead of increasing the *likeability* of a post, adding a link decreases the community *likeability* of a post to 76.8% (187/487; mean 2.34, SD 2.01 *likes*). This trend is even more pronounced in filtered threads that start with a question, where *likeability* falls from 72.27% (435/6063; mean 2.55, SD 2.47) to 63.2% (105/176; mean 1.47, SD 1.69) for replies with a link. This trend breaks only at the very end of the table: answers with relevant links (57/85, 71%; mean 1.94 *likes*) were still slightly more likable than average answers with links but were still less likable than an average reply to a post with a question (436/6063, 72.27%; mean 2.55). This interesting data indicate that the *liking* behavior of the originating user is different from the *liking* behavior of the whole community. We hypothesized that the likes of the target user were driven mostly by appreciation of the information and its relevance, whereas the *likes* of the community are driven more by compassion and acknowledgment of the effort to answer. In this situation, posts with links, which require more cognitive effort to consume before acknowledging, receive a lower share of *likes*, even if these posts look relevant. Unfortunately, this observation also means that community liking behavior cannot be considered a reliable indicator of a post’s value. An *H* test on *likes* from the community on comments with filtered threads further revealed that there was no significant difference (*H*=2.1; *P*=.10) between likes on comments with shared links and likes on comments with relevant shared links.

## Discussion

### Principal Findings

Survivors of OvCa and caregivers increasingly rely on OHCs for informational support [15,16]. Survivors of OvCa and caregivers can exchange information individualized to their needs on OvCa OHCs [15-18]. As a result of this information exchange, users often share resources through web links [17,18]. Survivors of OvCa and caregivers might not be health experts [62]; thus, it is vital to know if the resources shared on OvCa OHCs are relevant to their information needs. Research has examined resource sharing in OHCs in the past; however, there is a paucity of studies that look at the relevance of such resources. This study fills this gap by examining the relevance of shared resources on an OvCa OHC forum and extends prior research [22,23] by examining the association of resource relevance with different aspects of resource-sharing behavior. An in-depth understanding of resource-sharing behaviors associated with resource relevance can help find informative resources shared in OHCs. As expected, this study found that only half of all the shared resources were relevant to information needs. An analysis of different aspects of resource-sharing behavior suggests that resource behavior, including the purpose of sharing a resource and the type of resource, can be a reliable indicator of relevant shared resources, whereas explicit feedback of OHC users on a shared resource was not a reliable indicator of resource relevance.

### Resource Relevance

The results show that OvCa OHC peers can provide relevant resources related to the information needs of OvCa OHC users only half the time. This result does not indicate that users’ information needs from the initial post were not met. Rather, the results indicate that OvCa OHC users, who are survivors of OvCa and their caregivers (health care consumers who most of the time are not health care experts), might not be as efficient as we expected in finding relevant resources. For example, the user asks about the side effects of specific chemotherapy (altretamine), but the resource shared is pointing to the National Comprehensive Cancer Network guidelines [63], which contain general side effects from any chemotherapy but not specific to altretamine. In addition, from the shared resources, approximately 29.5% (52/176) of time the resources shared were partially relevant, which indicates that OvCa OHC users’ information needs are sometimes individualized and not addressed by generalized OvCa resources, such as patient education materials. This insight provides motivation for building a health resource recommender system that would individualize resources based on patients’ information needs and current disease trajectories.

This study found two important indicators for the topical relevance of shared resources: the types of shared resources and the purpose of shared resources. The findings related to the relationship between topical relevance and different resource-sharing behaviors complement and extend the study conducted by Zhang and Sun [22]. They studied the shared resources in the initial posts of a thread, whereas we investigated the shared resources in the comment posts to address the information needed in the initial post from the same thread. The fact that we studied the topical relevance of these resources and unveiled the association between resource-sharing behaviors and topical relevance may have the following benefits: (1) recognize the sources from which OvCa OHC users find relevant resources, (2) use resource-sharing behaviors to aggregate reliable shared resources in an OHC, and (3) recommend resources to OHC users with similar information needs so that they do not have to always rely on peer users.

### Resource Type and Resource Relevance

Exploration of resource type sharing revealed that NOCC users rely more on health consumer materials, including health news, health articles, and patient education resources, and less on health professional materials, such as academic literature (only 11/176, 6.3%). This could be as survivors of OvCa and caregivers often do not have adequate health literacy to understand health professional articles. On the other hand, patient materials targeted toward health consumers are probably more suitable [64]. However, when shared, academic literature was relevant to the information needed 73% (8/11) of the time. We assume that the high relevance of academic literature is because it can fulfill the complex information needs of OvCa OHC users.

NOCC peers also shared patient-generated materials, including patient blogs. Most of the time, patient blogs were meant to share their life journey and survival experiences with fellow users going through the OvCa journey. “*...*Is there anyone who has something similar?*...*” and “*...*Anyone out there survived against all odds for longer than 3 years before recurrence?” are some examples of information needed for which patient blogs were shared. A few times, patient blogs were also shared with the purpose *of staying connected* to the user who asked the question, as shown in the two following comments: “If XXX or you would like to connect with me more, I am at www.xxxblog.com” and “I recommend you go to my blog www.xxx.blogspot.com if you would like to stay in touch.” Thus, patient blogs are important resources that contain real patient experiences and provide a platform for connecting with fellow OHC users. Previous studies have found that forum users prefer narrative articles and user blogs over nonnarrative articles [65,66]. However, our study observed that patient blogs were shared only 4.5% (8/176) of the time. In addition, patient blogs shared with the purpose of *staying connected* were mostly partially relevant or irrelevant, as they were not targeted to answer OHC users’ specific information needs. We assume that the reason could be the complex and unique information needs of OvCa forum users. Hence, finding similar experiences is not always feasible. Therefore, only a few patient-generated articles were shared.

Prior research [22] observed that news articles were shared only 13% of the time, whereas we observed that news articles were shared many times (38/176, 21.6%). One of the reasons for this could be the rarity of OvCa. Survivors of OvCa and caregivers are looking for new treatments and information on clinical trials, and symptom management and health news are good resources for identifying these new findings. There were many shared resources pertaining to the news that included news on new clinical trials, the studied effect of OvCa medication, and recent studies about new treatments, which further clarified that survivors of OvCa and caregivers are eager to learn about new findings and treatments available for a cure.

### Resource Purpose and Resource Relevance

Zhang and Sun [22] studied the different purposes of resource sharing in a diabetes OHC. They studied resource sharing in initial posts, which were posted to share experiences, start discussions, and ask questions. However, we studied resource sharing in reply comments, which were intended to answer questions asked in the initial post of the thread. This is important as this study aimed to understand the relevance of shared resources, and questions asked in the initial post act as information needed against which the resource is shared.

Similar to Zhang et al [22], OvCa OHC users’ purposes for sharing resources include staying connected, providing further reading, subjects for discussion, and supporting arguments. However, a new category of *pointing to resources* was introduced in this study after the first round of annotator discussion. The *pointing to resources* category was added to handle cases where the purpose of the link was to provide available health resources (health institutions, search engines, or physicians) rather than providing direct reading material. We believe the reason for this category in our post is that Zhang et al [22] studied link sharing in initial posts, whereas this study focused on comment posts, where resources were shared to answer questions in the initial post. The *pointing to resources* purpose was used to answer questions regarding funding resources, clinical trials, and physicians’ listings. This category had the second-highest purpose of sharing resources. This finding also provides an important insight that patients on OvCa OHC require much advice on searching for treatment and funding resources. A chi-square test revealed that *pointing of resources* is associated with relevant articles and is an important indicator of relevant resources.

### User Reaction to Shared Resource and Resource Relevance

Previous studies have shown that the perceived credibility of a post increases if more people like the post or show gratitude toward it [67]. NOCC followed a similar pattern, with more *likes* on relevant links and fewer *likes* on nonrelevant and partially relevant links. However, these likes are different from average *like* behavior on links; thus, they are difficult to rely on as they are not significantly associated with shared resource relevance (Table 6). This can be inferred from users’ average *like* behavior, which changed from 1.41 to 1.74; therefore, is hardly noticeable and is not significant, as shown in Table 6. Sarma et al [36] observed that user *like* reactions were not useful in ranking informative comments on the Twitter platform [68]. In line with previous studies, despite higher coverage, the *like* reaction of the whole community might not be a reliable indicator of the overall usefulness of a resource in NOCC [69-71]. Furthermore, a *like* reaction from the original user with the question could serve as an indicator of resource relevance; however, the low coverage of these likes (at maximum 3/85, 4% for relevant resources) makes it difficult to use this source of feedback in practice for finding relevant resources.

### Future Work and Practical Implications

#### Health Care Educators

Our study observed that patient education materials were shared only 10.2% (18/176) of the time and were partially relevant or irrelevant 72% (13/18) of the time. This result informs health educators that patients often seek other materials to fulfill their information needs. One of the well-known education materials for patients with OvCa is the OvCa guidelines from the National Comprehensive Cancer Network. The guidelines were identified as partially relevant to the needs 86% (6/7) of the times shared. A possible reason could be that patient education materials have to be more personalized to satisfy an individual patient’s needs. This finding highlights a research gap for further improvement of patient education materials. For example, educational materials could include relevant patient case studies to be more personalized.

Another insight on patient educational materials is that patients with OvCa found more relevant documents from the news. This suggests that patient education materials can be updated with new facts and findings so that patients do not have to rely on external resources, which can potentially be misleading or untrustworthy.

#### OHC Administrators and Users

This study found that it is not informative on the relevance of shared resources to examine OHC users’ behaviors on the *like* button as feedback. This informs OHC forum administrators that a better and more informative feedback mechanism should be considered for OHCs. Our finding is also consistent with that of a recent study by Sarma et al [36], who found that forum user feedback in the form of *likes* is not enough to obtain informative feedback. A few examples of more informative feedback are the *helpful* button for shared resources, *best answer* button for initial post user feedback, and *best answer* button for forum moderator feedback. A more comprehensive study is required to understand better ways of obtaining user feedback on OHCs.

The study also reveals that there is an association between relevance and different aspects of resource-sharing behaviors. An important implication of this study could be the accumulation of a library of patients with OvCa and their caregivers. OHC administrators can collect the resources shared by users and provide a library of resources to users so that they can bookmark and use these resources for future use. Survivors of OvCa have different information needs at different stages of the disease and treatment trajectory [72]. A health article library with predefined information needs and topics could work as a frequently asked questions list, which patients can browse through to meet their specific needs.

#### Recommender Engine

It is a challenge to make informative content discoverable for patients with cancer. In addition, OvCa is a rare disease for which the internet has relatively fewer resources and experiences low quality [73]. Search engines help patients find information; however, their precision on the internet is low [16]. This study was conducted as part of our HELPeR project [74]. The goal of the HELPeR is to provide survivors of OvCa and caregivers with personalized health resources and reading materials. The study finding that relevant resources are shared only half of the time for a corresponding information need provides a motivation for the requirement of a recommender engine. It also provides insights that inform the types of resources to include and what roles these sources can fulfill; for instance, recommending more health news, health articles, and resources from health organizations that are frequently shared on NOCC. The finding of an association between relevance and resource-sharing behaviors reveals which user behaviors are reliable in determining the relevance of a resource. This can be used to automate data collection for training a machine learning–based recommender engine.

### Limitations

First, the relevance of a resource is based on topical relevance, which measures whether the provided resource addresses the corresponding question asked by the user. While checking for relevance, users’ knowledge level is not considered, which could play a major role in the relevance of a resource to an individual’s information need. For example, if a person with no medical background is provided with academic literature to fulfill their information needs, it may be difficult for them to understand the literature [20,75,76]. Similarly, other aspects of relevance, including the trustworthiness and clinical validity of the document, were not considered in this study [23,77]. Future work should combine the three aspects together to understand the relevance of a document, including topical relevance, users’ knowledge level, and resource trustworthiness.

Second, the study assumed OHC users’ information needs based on the questions asked by the user and the background information provided by the user within this post. The relevance of the shared resources is based on the user’s expressed information needs. This might not affect relevance if the actual information needed is different. For example, the user may not know how to express their information needs, or the user may not provide a proper context to fully understand their information needs.

Third, the study only analyzed 1 OvCa OHC; therefore, the results cannot be generalized to all OvCa OHCs. NOCC is a private and closely connected community; therefore, these results cannot be generalized to open OHCs such as WebMD [37] and question answering forums such as Yahoo Answers [78]. The study included data generated by the NOCC from 2010 to 2020 (10 years). However, NOCC contained only 909 threads during this period. This could be as OvCa is a rare cancer and is diagnosed in later stages. Hence, the study was performed on a very small data set. Future studies can include data from other OvCa OHCs to further improve the generalization and study scale.

Fourth, the study did not differentiate forum users based on their cancer stage and disease trajectory. We acknowledge that users in the later stages of the disease trajectory might have more expertise in handling the disease and treatment [76] and would thus have different views of relevant resources. However, as presented before, this is an inherent limitation of using a web-based forum, as users’ information about the disease trajectory, medications, and ongoing treatment might not be available.

In future work, we would also like to study how different types of information needs influence the relevance of resources. The type of information needed can range from early diagnosis to treatment decisions, disease management, and palliative care. This investigation can reveal specific cases or topics for which peers are unable to find relevant information. This will help in determining the simple and complex needs of OvCa OHC users and help us investigate which needs are still not fulfilled by OvCa OHC peers.

### Conclusions

Health professionals and clinicians are unable to support each need of survivors of OvCa and their caregivers. Health professionals provide survivors of OvCa with generic patient educational materials that are not sufficiently individualized to meet the needs specific to each patient. OHCs provide clinicians and researchers with a platform to observe the needs of survivors of OvCa and the resources that they rely on. In this study, we leveraged OHCs to investigate the resources that survivors of OvCa and their caregivers entrust to accomplish their and their peers’ information needs. Our study revealed that OHC users found more relevant resources from health news and health articles. Further investigation of OHC resource-sharing behavior revealed that direct evidence such as user reactions and TLDs were not enough to reveal the relevance of a resource, whereas implicit behavior, including types of resources shared and the purpose of resource sharing, had a direct association with resource relevance. The findings present implications and motivations for designing web-based recommender systems to support health information–seeking survivors of OvCa and caregivers. Subsequently, this resource collection will become part of our recommender system. Subsequent studies should further investigate how a resource’s relevance is influenced by the different types of information needs.

## Abbreviations

HELPeR: Health e-Librarian with Personalized Recommender
NOCC: National Ovarian Cancer Coalition
NOCC-RS: National Ovarian Cancer Coalition shared resource
OHC: online health community
OvCa: ovarian cancer
RQ: research question
TLD: top-level domain
